# Cancer mortality predictions for 2026 in selected Asian countries and Australia with focus on prostate cancer

**DOI:** 10.1097/CEJ.0000000000001013

**Published:** 2026-03-23

**Authors:** Claudia Santucci, Silvia Mignozzi, Fabio Levi, Matteo Malvezzi, Giovanni Corso, Eva Negri, Giorgio Gandaglia, Carlo La Vecchia

**Affiliations:** a Department of Clinical Sciences and Community Health, University of Milan; b Fondazione IRCCS Ca’ Granda Ospedale Maggiore Policlinico, Milan, Italy; c Department of Epidemiology and Health Services Research, Centre for Primary Care and Public Health (Unisanté), University of Lausanne, Lausanne, Switzerland; d Department of Medicine and Surgery, University of Parma, Parma; e Division of Breast Surgery, European Institute of Oncology (IEO), Istituto di Ricovero e Cura a Carattere Scientifico (IRCCS); f Department of Oncology and Hemato-Oncology, University of Milan, Milan; g Department of Medical and Surgical Sciences, University of Bologna, Bologna; h Department of Urology, Unit of Urology/Division of Oncology, Gianfranco Soldera Prostate Cancer Lab, Urological Research Institute (URI), IRCCS San Raffaele Scientific Institute, Milan, Italy

**Keywords:** Asia, Australia, cancer mortality, prediction, prostate cancer, trends

## Abstract

**Introduction:**

We estimated cancer mortality figures for 2026 in five major Asian countries and Australia, with a specific focus on prostate cancer.

**Methods:**

We computed country- and sex-specific annual age-standardized mortality rates (ASRs) for all cancers combined and for the 10 most common cancer sites, using data from the WHO and the United Nations Population Division up to 2022 or the most recent available year. We predicted figures for 2026 and estimated the number of avoided cancer deaths in 1994–2026.

**Results:**

Predicted mortality rates for all cancers combined in 2026 are favourable across all considered countries and in both sexes, with the largest declines expected in the Republic of Korea (−20.5% in males and −10.2% in females compared with 2020–2022). In 2026, the lowest predicted male rate is expected in the Philippines (72.1 per 100 000), and the highest one in Australia (92.3 per 100 000). Among females, the lowest predicted ASR (42.2 per 100 000) is in the Republic of Korea, whereas the highest one (74.1 per 100 000) in the Philippines. Trends are generally favourable for lung, stomach, colorectum, and other major neoplasms considered, except pancreas. Predicted prostate cancer mortality is favourable in all countries and across all age groups. Rates are expected to remain low in Hong Kong SAR, Japan, and the Republic of Korea, with ASRs below 4 per 100 000 males. Since the 1993 observed peak rate, an estimated 132 000 total cancer deaths were avoided in Hong Kong SAR, 75 000 in Israel, 1 366 000 in Japan, 720 000 in the Republic of Korea, 328 000 in Australia, and 102 000 among men in the Philippines.

**Conclusions:**

Declining cancer mortality is predicted in the countries considered. These trends largely reflect smoking cessation along with improvements in prevention, early detection, and treatment. However, substantial geographic disparities persist, highlighting the need for strengthened cancer control strategies, particularly in ageing populations.

## Introduction

Since 2018, we have regularly provided data on long-term trends in cancer mortality and short-term predictions for selected Australasian countries (Carioli *et al*., 2019). Declines in mortality rates have been observed for several major cancers, particularly stomach cancer in Japan and the Republic of Korea which maintain however comparably high rates, and male lung cancer. However, the absolute number of cancer deaths continues to rise due to population ageing (Santucci *et al*., 2024; Dee *et al*., 2025).

Here, we present updated mortality predictions for 2026 for Australia and five Asian countries: Hong Kong SAR, Israel, Japan, the Philippines, and the Republic of Korea. We also provide focussed analyses on prostate cancer, one of the most common cancers worldwide, whose growing epidemiological impact is driven by rising incidence, population ageing, and cross-country differences in early detection strategies and clinical management (Ko *et al*., 2025). In 2025, we published a companion paper on prostate cancer for Latin American countries (Mignozzi *et al*., 2026).

## Materials and methods

Official death certification data were derived from the WHO mortality database (WHO Statistical Information System, 2025) for all cancers combined and 10 major cancer sites, that is, stomach, colorectum, pancreas, lung, breast, uterus (cervix and corpus), ovary, prostate, bladder, and leukaemias. We retrieved both observed and predicted resident population estimates from the United Nations database (UN, 2024). We included data for Australia (1970–2023) and five Asian countries with over 7 million inhabitants and with both high coverage and data quality (WHO Statistical Information System, 2020), that is, Hong Kong SAR (1970–2021), Israel (1975–2022), Japan (1970–2021), the Philippines (1992–2019), and the Republic of Korea (1985–2022). No valid data were available from the WHO database on other Asian countries with population over 7 million. Sex-specific mortality rates were estimated for each cancer site by 5-year age class (0–4 to 85+ years) and calendar year. Annual and quinquennial age-standardized mortality rates (ASRs) per 100 000 individuals were calculated based on the world standard population. For prostate cancer, truncated ASRs were additionally computed for the 50–59, 60–69, 70–79, and ≥80 age groups. Trends in ASRs over time were analysed through joinpoint regression analysis (Kim *et al*., 2000).

We derived predicted figures for 2026 and the corresponding 95% prediction intervals by applying a logarithmic Poisson joinpoint regression model to the number of deaths in each five-year age group. To estimate predicted age-specific numbers of deaths we fitted a linear regression model to the death counts of each age group over the most recent segment identified by the joinpoint model. We computed age-specific and age-standardized death rates for 2026 and their corresponding 95% prediction intervals using the predicted age-specific death counts and the corresponding predicted population data. The number of deaths avoided during 1994–2026 was estimated by comparing the observed and expected deaths based on the 1993 age-specific rate, as the peak of cancer mortality was observed around the year 1993 in all countries, except for the Philippines. For the Philippines, where mortality rates peaked around 2000, avoided deaths were estimated for the period 2001–2026.

Statistical analyses were performed using the software R version 4.5.0 (R Core Team, 2025, R Foundation for Statistical Computing, Vienna, Austria), SAS version 9.4 (SAS Institute Inc., Cary, NC, USA), and Joinpoint Regression Program version 4.9.1.0 (Statistical Methodology and Applications Branch, Surveillance Research Program, National Cancer Institute).

## Results

Table 1 gives the number of predicted deaths and the mortality rates for all cancers combined and the most common cancers for the year 2026 and comparison figures for 2020–2022 among males, with 95% prediction intervals and the percentage differences in rates. Table 2 gives the corresponding figures for females. Figure 1 shows the ASR from all cancers combined in bar plots for each country, according to sex, in the 2020–2022 period and the predicted rates for 2026, with the corresponding 95% prediction intervals.

**Table 1:** Number of predicted deaths and mortality rates for the year 2026 and comparison figures for 2020–2022 among males, with 95% prediction intervals and the percentage differences

	Mean annual number of deaths 2020–2022^a^	Predicted number of deaths 2026 (95% PI)	Observed ASR 2020–2022^a^	Predicted ASR 2026 (95% PI)	Δ% 2026 vs. 2020–2022^a^
Hong Kong SAR					
Stomach	375	340 (292–387)	4.03	3.00 (2.40–3.59)	−25.56
Colorectum	1379	1550 (1446–1658)	14.67	14.83 (13.65–16.00)	1.09
Pancreas	466	550 (506–602)	5.47	6.12 (5.59–6.64)	11.88
Lung	2574	2660 (2497–2813)	27.88	24.41 (22.54–26.28)	−12.45
Prostate	501	560 (493–620)	4.16	3.91 (3.47–4.35)	−6.01
Bladder	157	160 (137–191)	1.39	1.26 (1.00–1.52)	−9.35
Leukaemias	208	180 (151–214)	2.71	1.99 (1.26–2.72)	−26.57
All cancers	8879	9170 (8879–9454)	98.82	87.92 (84.35–91.48)	−11.03
Israel					
Stomach	252	260 (218–293)	3.90	3.56 (3.01–4.11)	−8.72
Colorectum	657	630 (562–704)	9.69	8.19 (7.20–9.18)	−15.48
Pancreas	520	560 (510–618)	8.12	7.70 (6.80–8.60)	−5.17
Lung	1221	1190 (1099–1275)	19.34	16.02 (14.65–17.39)	−17.17
Prostate	492	510 (457–570)	6.05	5.70 (5.09–6.31)	−5.79
Bladder	334	350 (296–398)	4.34	3.97 (3.42–4.52)	−8.53
Leukaemias	274	270 (234–301)	4.00	3.43 (2.93–3.93)	−14.25
All cancers	6227	6690 (6470–6916)	92.45	86.67 (83.54–89.79)	−6.25
Japan					
Stomach	27 482	23 280 (22 332–24 224)	11.53	7.84 (7.13–8.54)	−32.00
Colorectum	29 124	29 720 (28 791–30 640)	13.76	12.65 (12.11–13.20)	−8.07
Pancreas	19 106	21 290 (20 761–21 811)	9.08	9.44 (9.14–9.74)	3.96
Lung	53 260	51 710 (49 861–53 565)	22.06	18.18 (17.22–19.14)	−17.59
Prostate	12 988	13 320 (12 753–13 890)	4.03	3.74 (3.56–3.92)	−7.20
Bladder	6339	6420 (6021–6818)	2.20	1.94 (1.79–2.10)	−11.82
Leukaemias	5508	5730 (5510–5947)	2.85	2.49 (2.27–2.71)	−12.63
All cancers	228 894	226 600 (221 331–231 866)	98.87	83.97 (80.32–87.62)	−15.07
Philippines					
Stomach	732	970 (871–1070)	2.40	1.92 (1.72–2.12)	−20.00
Colorectum	3284	5010 (4785–5237)	10.72	9.69 (9.26–10.12)	−9.61
Pancreas	735	1100 (1035–1154)	2.40	2.11 (1.99–2.23)	−12.08
Lung	4878	6290 (6018–6561)	16.55	12.53 (11.99–13.08)	−24.29
Prostate	2459	3790 (3637–3934)	9.74	9.05 (8.61–9.48)	−7.08
Bladder	229	340 (304–368)	0.80	0.70 (0.63–0.77)	−12.50
Leukaemias	1259	1780 (1681–1876)	3.32	3.10 (2.92–3.27)	−6.63
All cancers	25 369	36 790 (36 100–37 481)	82.43	72.05 (70.54–73.55)	−12.59
Republic of Korea					
Stomach	4732	3920 (3633–4209)	8.91	5.82 (5.33–6.32)	−34.68
Colorectum	5376	5380 (5144–5621)	10.08	8.28 (7.88–8.69)	−17.86
Pancreas	3622	4120 (3890–4339)	6.80	6.39 (6.02–6.77)	−6.03
Lung	13 831	13 970 (13 364–14 582)	24.71	19.54 (18.71–20.37)	−20.92
Prostate	2312	2510 (2323–2686)	3.95	3.21 (2.97–3.45)	−18.73
Bladder	1198	1250 (1152–1351)	2.09	1.68 (1.53–1.82)	−19.62
Leukaemias	1136	1170 (1071–1262)	2.51	1.96 (1.64–2.28)	−21.91
All cancers	51 903	52 330 (50 887–53 763)	96.94	77.12 (74.85–79.40)	−20.45
Australia					
Stomach	1019	750 (684–813)	2.84	2.56 (2.31–2.81)	−9.86
Colorectum	4066	3030 (2885–3181)	10.91	9.59 (9.05–10.12)	−12.10
Pancreas	2444	1990 (1888–2095)	6.72	6.65 (6.29–7.01)	−1.04
Lung	6692	5230 (4980–5485)	17.80	16.31 (15.52–17.11)	−8.37
Prostate	4917	4040 (3873–4209)	10.13	9.47 (9.05–9.90)	−6.52
Bladder	1081	760 (674–845)	2.30	1.90 (1.68–2.11)	−17.39
Leukaemias	1656	1350 (1276–1429)	4.15	3.83 (3.50–4.15)	−7.71
All cancers	38 241	30 850 (30 197–31 502)	98.93	92.31 (90.19–94.43)	−6.69

ASR, age-standardized mortality rate; PI, prediction interval.

a 2020–2021 for Hong Kong SAR and Japan, 2015–2019 for the Philippines.

**Table 2:** Number of predicted deaths and mortality rates for the year 2026 and comparison figures for 2020–2022 among females, with 95% prediction intervals and the percentage differences

	Mean annual number of deaths 2020–2022^a^	Predicted number of deaths 2026 (95% PI)	Observed ASR 2020–2022^a^	Predicted ASR 2026 (95% PI)	Δ% 2026 vs. 2020–2022^a^
Hong Kong SAR					
Stomach	247	270 (232–304)	2.30	2.28 (1.88–2.68)	−0.87
Colorectum	1010	1110 (1016–1202)	8.58	7.79 (6.98–8.61)	−9.21
Pancreas	386	420 (380–460)	3.49	3.49 (3.14–3.84)	0.00
Lung	1400	1450 (1333–1574)	12.65	11.35 (10.26–12.45)	−10.28
Breast	771	940 (854–1022)	8.82	9.88 (8.93–10.84)	12.02
Uterus	348	410 (370–449)	4.03	4.47 (4.01–4.93)	10.92
Ovary	219	270 (236–300)	2.65	3.00 (2.59–3.42)	13.21
Bladder	62	60 (41–74)	0.40	0.28 (0.16–0.40)	−30.00
Leukaemias	148	150 (126–173)	1.75	1.34 (0.63–2.05)	−23.43
All cancers	6470	6870 (6654–7084)	61.95	56.15 (53.73–58.56)	−9.36
Israel					
Stomach	170	180 (144–220)	2.26	2.34 (1.91–2.77)	3.54
Colorectum	612	540 (474–602)	7.01	5.59 (4.83–6.35)	−20.26
Pancreas	468	480 (420–533)	5.62	5.11 (4.43–5.80)	−9.07
Lung	652	670 (593–741)	8.51	7.75 (6.80–8.70)	−8.93
Breast	1031	1030 (938–1122)	13.87	11.88 (10.47–13.28)	−14.35
Uterus	289	290 (251–326)	4.06	3.90 (3.40–4.40)	−3.94
Ovary	297	290 (242–329)	4.12	3.48 (2.83–4.13)	−15.53
Bladder	86	60 (44–83)	0.90	0.66 (0.48–0.85)	−26.67
Leukaemias	217	240 (211–278)	2.50	2.65 (2.24–3.06)	6.00
All cancers	5857	6230 (5967–6500)	71.58	67.50 (64.44–70.55)	−5.70
Japan					
Stomach	14 488	12 670 (12 167–13 169)	4.49	2.83 (2.51–3.16)	−36.97
Colorectum	25 154	26 330 (25 704–26 955)	8.08	7.63 (7.27–7.98)	−5.57
Pancreas	19 020	22 150 (21 574–22 716)	6.09	6.66 (6.35–6.97)	9.36
Lung	22 634	24 250 (23 495–24 994)	6.79	6.33 (5.95–6.70)	−6.77
Breast	14 726	15 030 (14 517–15 549)	8.64	7.87 (7.43–8.30)	−8.91
Uterus	6813	7180 (6897–7471)	4.22	4.51 (4.28–4.74)	6.87
Ovary	5171	5500 (5234–5767)	3.19	3.27 (3.07–3.47)	2.51
Bladder	2966	3290 (3143–3446)	0.64	0.67 (0.62–0.72)	4.69
Leukaemias	3546	3430 (3254–3615)	1.49	1.11 (0.95–1.27)	−25.50
All cancers	164 353	169 120 (166 149–172 092)	58.48	53.41 (51.44–55.38)	−8.67
Philippines					
Stomach	536	730 (677–792)	1.48	1.26 (1.17–1.36)	−14.86
Colorectum	2673	4110 (3948–4269)	7.31	6.99 (6.72–7.26)	−4.38
Pancreas	823	1240 (1161–1310)	2.29	2.10 (1.97–2.22)	−8.30
Lung	2327	3460 (3343–3581)	6.47	5.87 (5.66–6.08)	−9.27
Breast	7053	10 960 (10 632–11 280)	18.96	18.58 (18.01–19.14)	−2.00
Uterus	2810	4230 (4023–4430)	7.49	7.09 (6.75–7.43)	−5.34
Ovary	1573	2508 (2408–2608)	4.23	4.26 (4.09–4.43)	0.71
Bladder	113	160 (139–181)	0.32	0.28 (0.25–0.32)	−12.50
Leukaemias	1162	1620 (1530–1701)	2.95	2.73 (2.59–2.87)	−7.46
All cancers	27 532	43 600 (42 941–44 262)	74.26	74.05 (72.91–75.19)	−0.28
Republic of Korea					
Stomach	2570	2110 (1943–2274)	3.62	2.33 (2.01–2.65)	−35.64
Colorectum	4082	4190 (3990–4392)	5.19	4.44 (4.16–4.72)	−14.45
Pancreas	3389	3880 (3631–4131)	4.56	4.53 (4.24–4.82)	−0.66
Lung	4889	5080 (4895–5268)	6.33	5.31 (5.04–5.57)	−16.11
Breast	2770	2930 (2787–3064)	5.53	5.72 (5.44–6.01)	3.44
Uterus	1317	1350 (1246–1460)	2.42	2.32 (2.08–2.55)	−4.13
Ovary	1397	1450 (1357–1536)	2.48	2.37 (2.18–2.55)	−4.44
Bladder	377	410 (365–459)	0.41	0.36 (0.31–0.41)	−12.20
Leukaemias	830	850 (762–939)	1.54	1.16 (0.84–1.48)	−24.68
All cancers	32 499	34 610 (33 686–35 539)	47.01	42.23 (40.83–43.64)	−10.17
Australia					
Stomach	554	400 (347–445)	1.32	1.16 (0.99–1.34)	−12.12
Colorectum	3667	2800 (2681–2928)	8.02	7.39 (6.99–7.80)	−7.86
Pancreas	2252	1950 (1870–2035)	5.10	5.22 (4.96–5.48)	2.35
Lung	5035	3950 (3781–4120)	12.14	10.91 (10.34–11.48)	−10.13
Breast	4203	3210 (3068–3357)	11.15	10.19 (9.68–10.7)	−8.61
Uterus	1144	940 (878–999)	3.21	3.29 (3.06–3.52)	2.49
Ovary	1403	1080 (995–1168)	3.58	3.32 (2.98–3.65)	−7.26
Bladder	433	320 (274–358)	0.73	0.62 (0.52–0.72)	−15.07
Leukaemias	1053	810 (759–866)	2.32	2.05 (1.81–2.30)	−11.64
All cancers	29 745	24 000 (23 539–24 453)	69.72	66.15 (64.79–67.50)	−5.12

ASR, age-standardized mortality rate; PI, prediction interval.

a 2020–2021 for Hong Kong SAR and Japan, 2015–2019 for the Philippines.

**Fig. 1:**
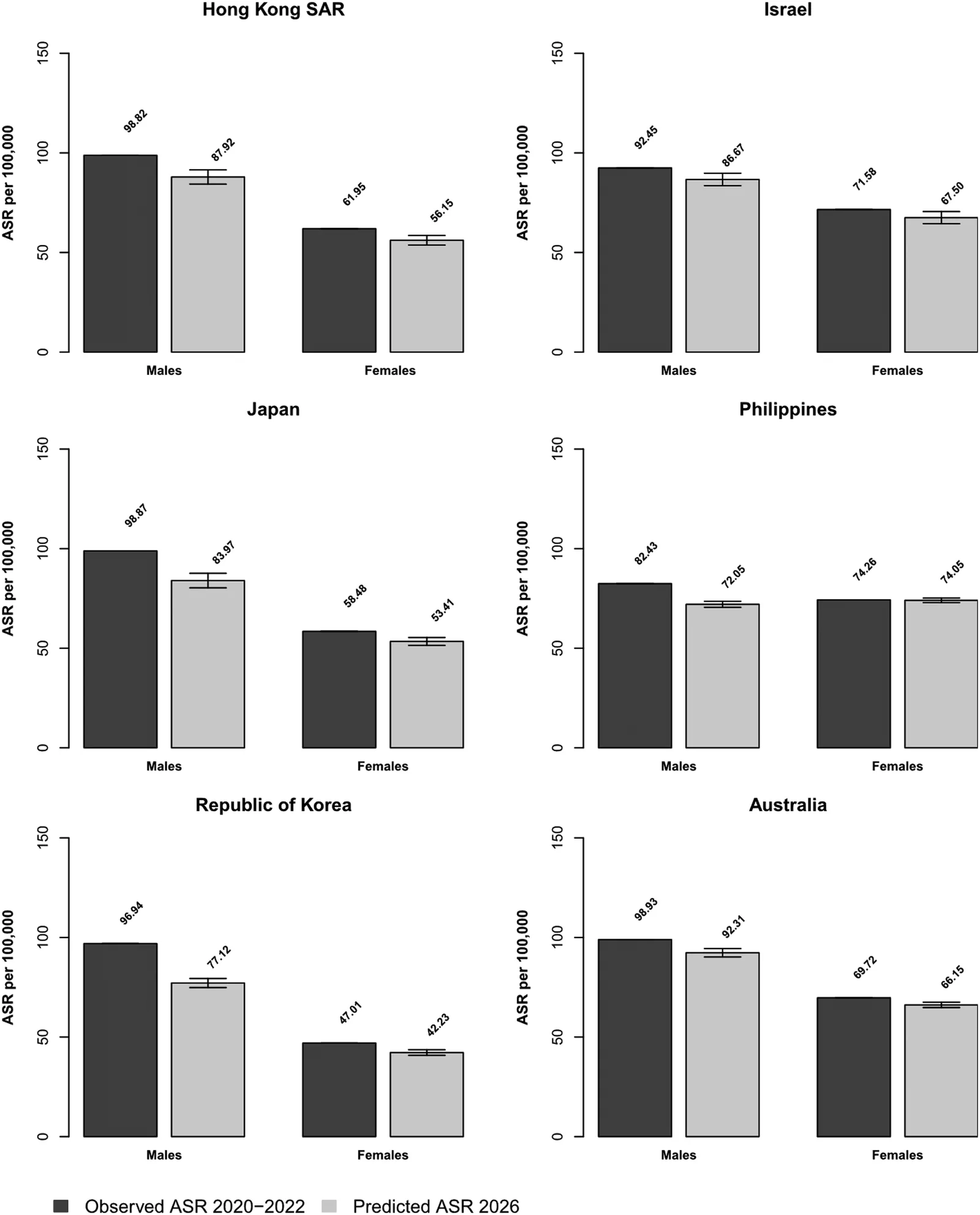
Bar plots of age-standardized mortality rates (ASR) per 100 000 males and females at all ages for 2020–2022 and predicted ASR for 2026, with 95% prediction intervals, for all cancers combined in Hong Kong SAR, Israel, Japan, the Philippines, the Republic of Korea, and Australia.

Among males, cancer mortality from all cancers combined is predicted to decrease in 2026 in all considered countries, with reductions ranging from −6.3% vs. 2020–2022 in Israel to –20.5% in the Republic of Korea. Compared with 2020–2022, a slight increase in the number of male cancer deaths is expected in 2026 in all countries considered, except Australia where a decrease is predicted, and the Philippines, where, conversely, a larger increase is expected. The lowest 2026 male ASR for all cancers combined was predicted in the Philippines (72.1/100 000) and the highest one in Australia (92.3/100 000). Lung cancer is predicted to remain the leading cause of cancer deaths in most countries considered, with rates ranging from 12.5/100 000 in the Philippines to 24.4/100 000 males in Hong Kong SAR.

A downward trend in overall cancer mortality among females is predicted in all countries except the Philippines, where the 2026 ASR is predicted to remain stable with the highest rate among the considered countries (74.1/100 000 females, −0.28%). The highest decrease is in the Republic of Korea (−10.2% vs. 2020–2022), while the lowest one is expected in Australia (−5.1%). The number of female cancer deaths is projected to increase in 2026 in all selected countries, except Australia, with more substantial rises in the Philippines. The lowest female mortality rate was predicted in the Republic of Korea (42.2/100 000), whereas the highest rate in the Philippines (74.1/100 000). Breast cancer is expected to be the leading cause of female cancer death in 2026, with ASRs ranging from 5.7/100 000 in the Republic of Korea to 18.6/100 000 in the Philippines, except in Hong Kong SAR and Australia were lung cancer ranked first with rates of 11.4/100 000 and 10.9/100 000, respectively.

Figure 2 shows quinquennial ASRs per 100 000 from 1970 to 2022 and predicted ASRs for 2026, with 95% prediction intervals, for all cancers combined by sex. Hong Kong SAR and Australia recorded the highest rates in earlier quinquennia, but they were the first countries to experience a decline in overall male cancer mortality, beginning respectively in the mid-1980s and mid-1970s. In Japan and Israel, male ASRs began to decline in the mid to late 1990s, whereas in the Philippines and the Republic of Korea downward trends have emerged since the 2000s. Female cancer mortality rates declined during the whole period in Japan, whereas in Australia and Hong Kong SAR, the downward trend began between 1980 and 1990, and in Israel from 1990. Conversely, rates in the Philippines increased substantially from 1990 onwards, but stabilized after 2015–2019.

**Fig. 2:**
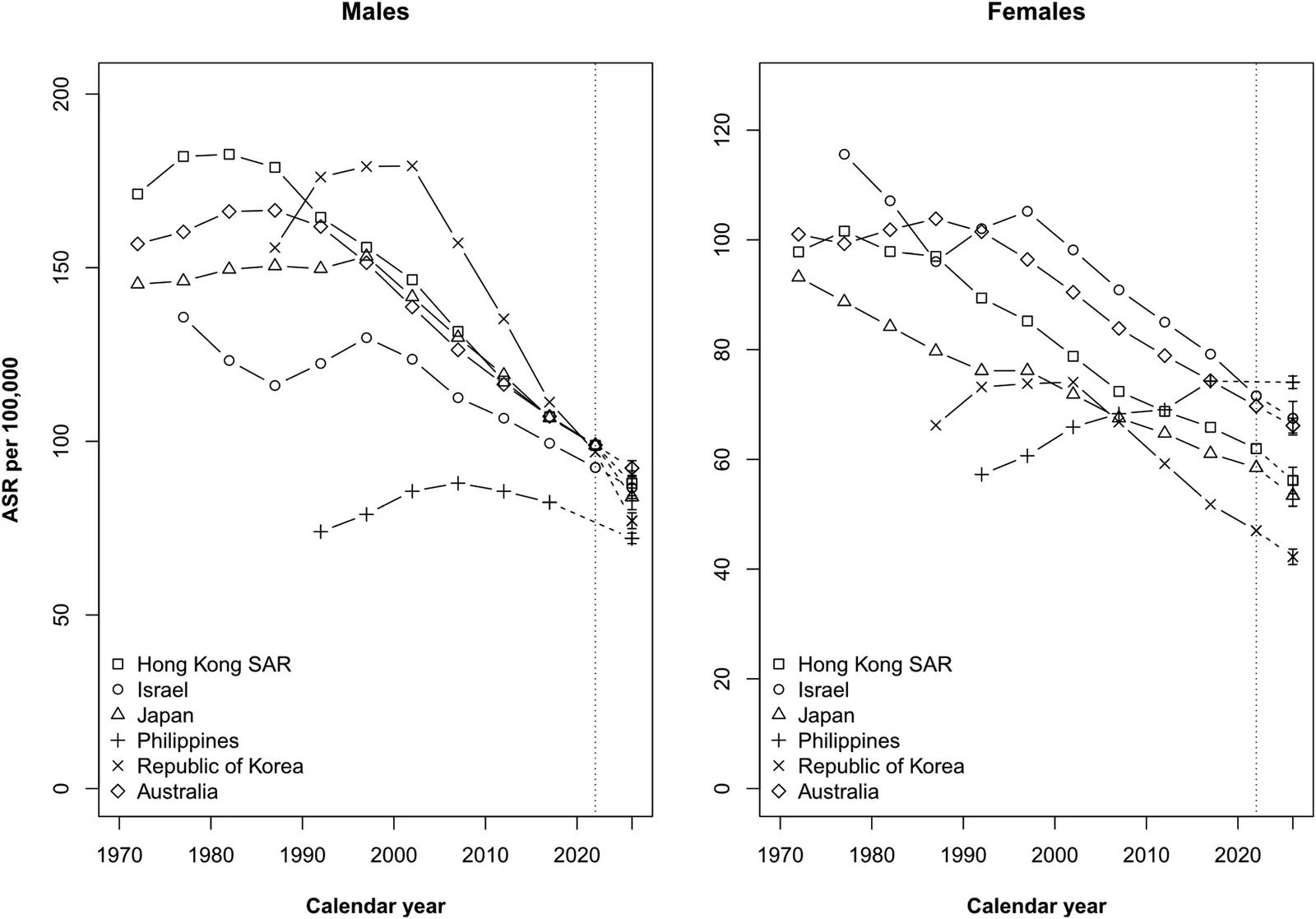
Age-standardized mortality rates (ASRs) per 100 000 from 1970 to 2022 and predicted ASRs for 2026, with 95% prediction intervals, for all cancers combined by sex in Hong Kong SAR, Israel, Japan, the Philippines, the Republic of Korea, and Australia.

Figure 3 reports quinquennial ASR for each cancer site, for males and females. Since 1970, male stomach cancer ASRs have been declining steadily across all the studied countries. In Japan and the Republic of Korea stomach cancer had the highest ASR until the mid-1990s, when – due to their substantial declines – the leading cause of cancer death became lung cancer. Mortality rates from male colorectal cancer began to decline in Israel between the mid-1980s and mid-1990s, and in the Republic of Korea during the mid-2000s. A downward trend also began in Australia in the late 1980s. Colorectal mortality remained stable among males in Japan, while in the Philippines it showed an unfavourable trend throughout the entire period, but a decrease is predicted for 2026. In all countries, pancreatic cancer mortality trends among males remained stable or increased over the study period, with unfavourable predictions for 2026 in Hong Kong SAR and Japan. In Australia, male lung cancer ASRs began to decline around 1980, in Hong Kong SAR and Japan, between the mid-1990s and early 2000s. The decline started later in the mid-2000s in Israel, the Philippines, and the Republic of Korea. In most countries, prostate cancer mortality remained stable or slightly increased. Mortality from bladder cancer and leukaemias showed modest declines or stable trends in most countries.

**Fig. 3:**
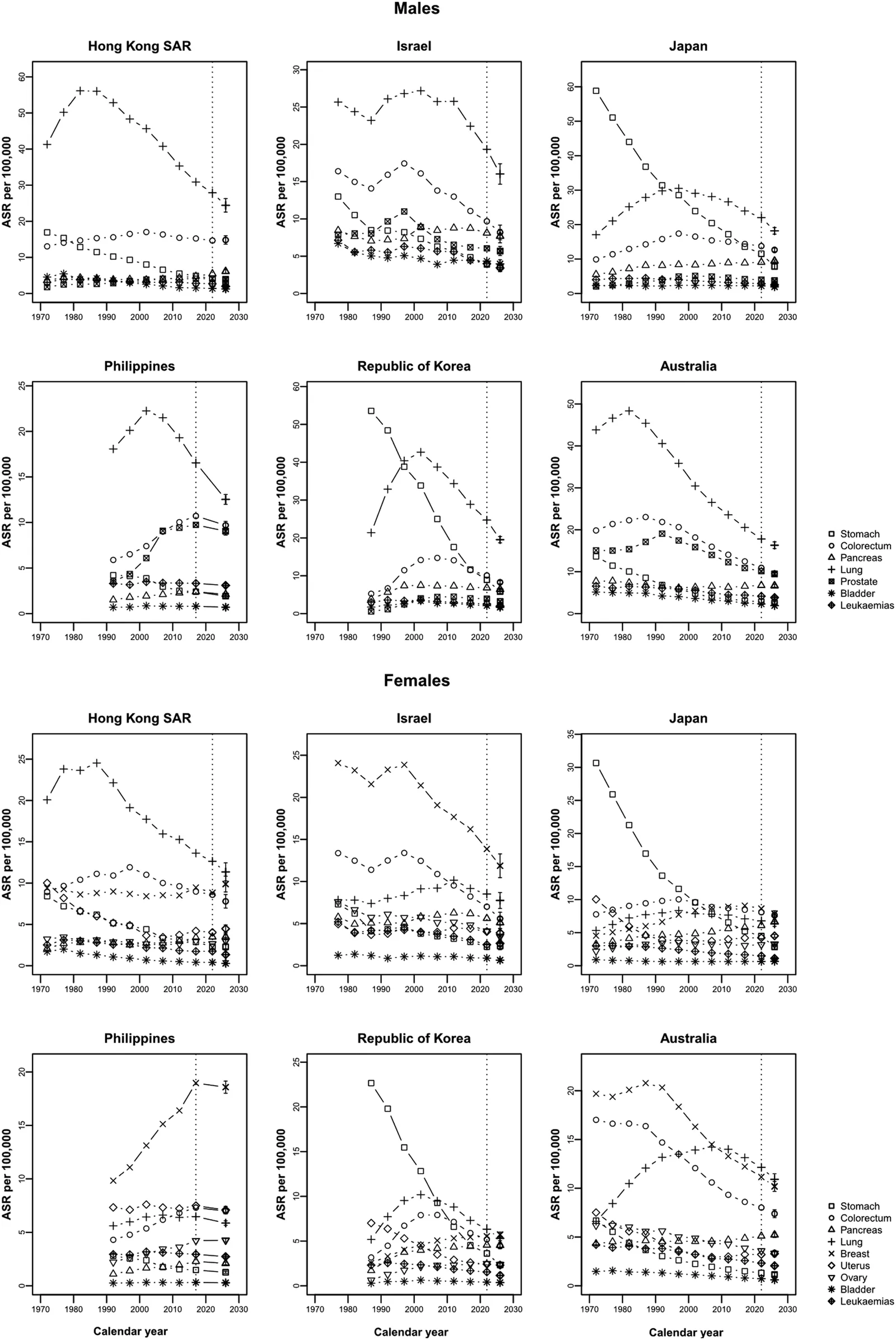
Age-standardized mortality rates (ASRs) per 100 000 from 1970 to 2022 and predicted ASRs for 2026, with 95% prediction intervals, for selected cancer sites, according to sex and country.

Among females, stomach cancer ASRs showed favourable trends over the entire period in all countries. In Japan and the Republic of Korea, stomach cancer remained the leading cause of cancer death until the mid-2000s. Colorectal cancer mortality among females began to decline in the mid-1980s in Australia, in the mid-1990s in Hong Kong SAR, Israel, and Japan, and in the 2000s in the Republic of Korea. In contrast, the Philippines experienced a slight upward trend, although with a favourable predicted rate for 2026. Female mortality from pancreatic cancer remained stable or increased across all countries. Lung cancer mortality increased in the early years before beginning to decline around 1990 in Hong Kong SAR, which had the highest rates of all countries considered studied. In Japan, the Philippines, the Republic of Korea, and Australia, this downward trend started around 2000, whereas Israeli females experienced a gradual increase in mortality rates from 1990 to 2015, followed by a decline. Around 1990, breast cancer ASRs began to decline appreciably in Israel and Australia, while in Japan they levelled off from 2015 onwards. Conversely, there was a moderate overall increase in breast cancer mortality, with unfavourable rates projected for Hong Kong SAR and the Republic of Korea in 2026. In the Philippines, mortality from breast cancer increased steadily but is predicted to decrease slightly up to 2026. Uterine cancer mortality trends have been generally stable or have shown favourable patterns across all countries. However, in recent years, an increase has been observed in Hong Kong SAR and Japan. Declining trends were recorded for ovarian cancer in Australia and Israel since 1970. Other countries reported stable or slightly increasing trends, with a predicted levelling off in recent years, except for Hong Kong SAR and Japan, where an increase is predicted for the 2026. Trends in female mortality from bladder cancer and leukaemias were stable or decreasing in most countries. However, an unfavourable ASR for bladder cancer is expected in Japan by 2026.

Table 3 gives ASRs from prostate cancer at all ages and according to different age groups (50–59, 60–69, 70–79, and 80+ years) in 2015–2019 and 2020–2022, along with predicted figures for 2026. Declines are predicted at all ages in 2026 vs. 2020– 2022, ranging from −5.8% in Israel to −18.7% in the Republic of Korea. The highest predicted ASRs at all ages were expected in the Philippines and Australia (above 9 per 100 000), while the lowest one is predicted in the Republic of Korea (3.2/100 000). Decreases are also expected in all age groups considered, except for Israeli males aged 60–69 (+10.0% vs. 2020–2022), Japanese males aged 70–79 (+13.3%), and Philippine males aged 80 and over (+10.5% vs. 2015–2019).

**Table 3:** Age-standardized prostate cancer mortality rates per 100 000 males in selected Australasian countries, overall and by age group, during 2015–2019 and 2020–2022, with predicted rates and prediction intervals for 2026, along with percentage differences between 2020–2022 and 2026

	ASR 2015–2019	ASR 2020–2022^a^	Predicted ASR 2026 (95% PI)	Δ% 2026 vs. 2020–2022^a^
Hong Kong SAR				
All ages	4.07	4.16	3.91 (3.47–4.35)	−6.01
50–59 years	1.43	1.97	1.71 (0.22–3.19)	−13.20
60–69 years	11.61	10.79	9.70 (7.52–11.88)	−10.10
70–79 years	44.05	48.17	46.09 (38.18–54.00)	−4.32
80+ years	179.87	176.53	168.25 (138.49–198.01)	−4.69
Israel				
All ages	6.19	6.05	5.70 (5.09–6.31)	−5.79
50–59 years	2.48	1.96	1.51 (0.00–3.30)	−22.96
60–69 years	15.99	17.37	19.11 (13.82–24.40)	10.02
70–79 years	70.07	67.27	66.13 (57.76–74.49)	−1.69
80+ years	272.77	262.88	224.82 (187.25–262.39)	−14.48
Japan				
All ages	4.16	4.03	3.74 (3.56–3.92)	−7.20
50–59 years	1.42	1.38	0.81 (0.36–1.26)	−41.30
60–69 years	11.20	10.19	5.89 (4.66–7.11)	−42.20
70–79 years	44.20	43.08	48.81 (44.69–52.92)	13.30
80+ years	190.52	188.06	176.82 (167.09–186.56)	−5.98
Philippines				
All ages	9.74		9.05 (8.61–9.48)	−7.08
50–59 years	4.62		4.33 (3.68–4.98)	−6.28
60–69 years	28.56		25.15 (23.27–27.02)	−11.94
70–79 years	118.05		90.96 (84.74–97.18)	−22.95
80+ years	369.56		408.26 (371.8–444.72)	10.47
Republic of Korea				
All ages	4.02	3.95	3.21 (2.97–3.45)	−18.73
50–59 years	1.25	1.10	1.06 (0.34–1.78)	−3.64
60–69 years	8.27	7.35	6.39 (5.56–7.22)	−13.06
70–79 years	42.62	40.88	32.87 (27.61–38.13)	−19.59
80+ years	202.72	208.83	165.74 (150.14–181.33)	−20.63
Australia				
All ages	10.86	10.13	9.47 (9.05–9.90)	−6.52
50–59 years	4.52	3.86	3.29 (2.04–4.53)	−14.77
60−69 years	29.50	25.75	24.44 (21.36–27.52)	−5.09
70–79 years	109.22	101.21	99.04 (90.00–108.08)	−2.14
80+ years	507.87	489.94	445.69 (423.96–467.41)	−9.03

ASR, age-standardized mortality rate; PI, prediction interval.

a 2020–2021 for Hong Kong SAR and Japan, 2015–2019 for the Philippines.

Figure 4 shows the estimated number of avoided cancer deaths in males and females between 1994 (2001 for the Philippines) and 2026, assuming the age-specific rates in 1993 (2000 for the Philippines) as constant. Over the period considered, a total of 132 000 avoided cancer deaths were estimated in Hong Kong SAR, 75 000 in Israel, 1 366 000 in Japan, 720 000 in the Republic of Korea, 328 000 in Australia, and 102 000 among Philippine males.

**Fig. 4:**
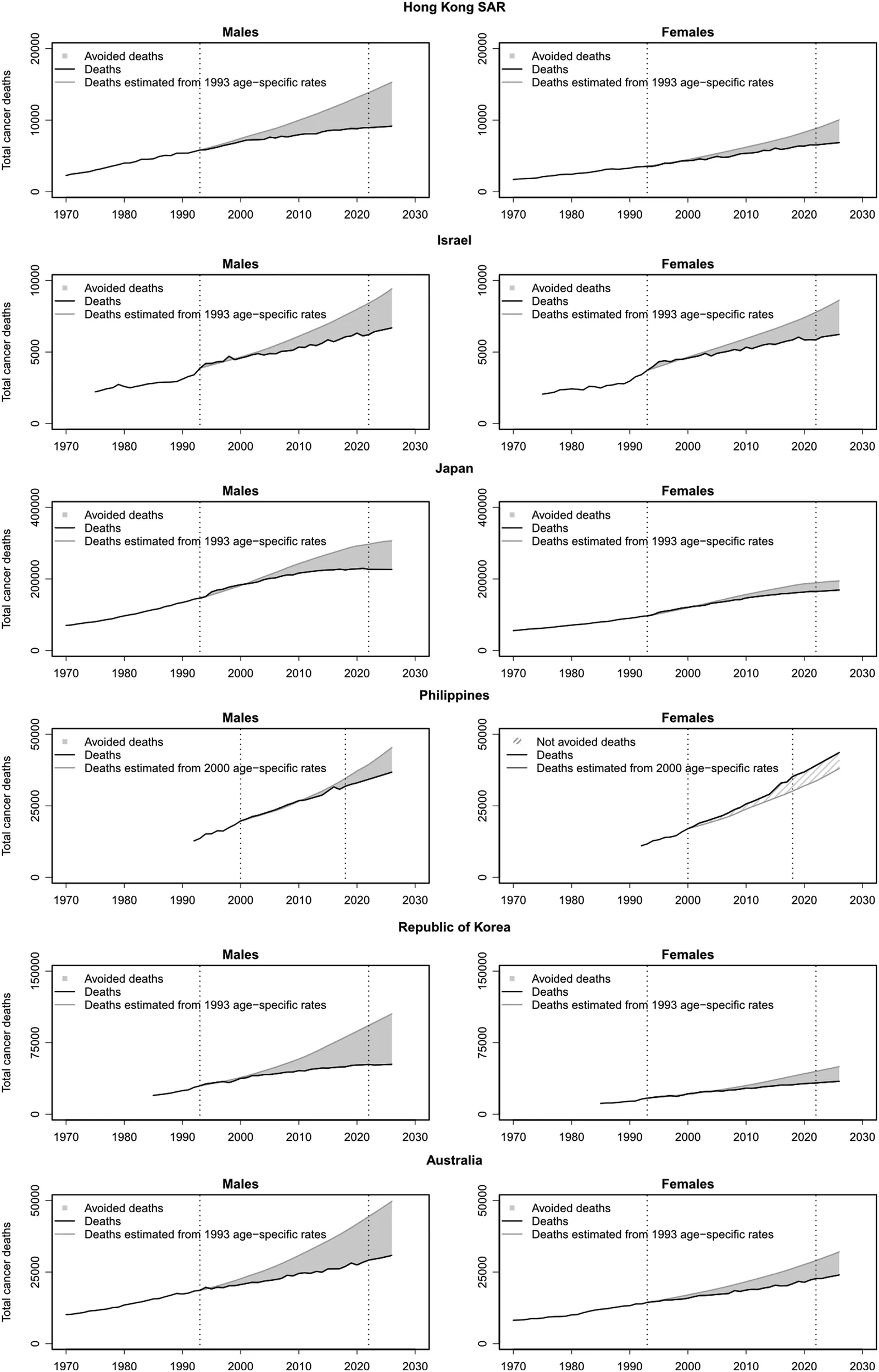
Total avoided cancer deaths by sex and country, between the peak mortality year (1993; 2000 for the Philippines) and 2026; observed and predicted numbers of cancer deaths from 1993 to 2026; and estimated total cancer deaths based on age-specific peak mortality rates.

## Discussion

The overall decline in age-standardized cancer mortality predicted for 2026 across the six Australasian countries providing valid data from the WHO database confirms the favourable long-term trends observed over recent decades, despite persistent geographic heterogeneity. However, as previously reported in Europe and the USA (Santucci *et al*., 2026; Siegel *et al*., 2026), the absolute number of cancer deaths continues to increase, largely driven by population ageing, highlighting the growing demographic pressure on cancer control systems.

### Prostate cancer

Prostate cancer mortality shows marked geographical heterogeneity across Australasia. Australia and the Philippines display the highest mortality rates, broadly comparable to those observed in Europe and North America (Ferlay *et al*., 2024), whereas Hong Kong SAR, Israel, and particularly Japan, and the Republic of Korea maintain some of the lowest rates worldwide (Jahn *et al*., 2015; Bergengren *et al*., 2023).

Australian mortality patterns closely resemble those of Western countries, consistent with its predominantly European ancestry, lifestyle factors such as diet, physical activity patterns and healthcare seeking behaviours, the adoption of prostate-specific antigen testing in the early detection setting, and the long-standing availability of advanced diagnostic and therapeutic approaches (Bergengren *et al*., 2023; Shevach and Cooney, 2025). In contrast, the low prostate cancer mortality in East Asian countries likely reflects the historically low incidence, likely due to a combination of genetic factors, lifestyle and environmental exposures (Jahn *et al*., 2015; Ito *et al*., 2025). Among these, diets rich in phytoestrogens – such as genistein, found in soy and soybean oil and consumed in particularly high amounts in Japanese and Korean populations – may partly explain the low rates reported across Asia (He *et al.*, 2015). However, incidence is rising with increasing westernization (Ko *et al*., 2025; Li *et al*., 2025).

The persistently unfavourable mortality in the Philippines represents a notable exception within the region. Evidence from other non-Western settings indicates that low awareness of prostate cancer and limited uptake of PSA-based early detection policies are strongly associated with late-stage diagnosis and poorer outcomes (Mohamed *et al*., 2025). These factors, together with restricted access to timely diagnosis and modern treatments, are likely to play a major role in sustaining high prostate cancer mortality in the Philippines, where rates are now approaching those observed in Western countries (Martin *et al*., 2013; Vergara *et al*., 2023).

Predicted prostate cancer mortality rates for 2026 indicate modest declines in most countries, consistent with improvements in disease management previously documented in high-income settings. Age-specific analyses show that the largest proportional reductions are expected among males aged 50–69 years, while declines are smaller at older ages, where prostate cancer mortality remains highest. This might also be related to the increased awareness of prostate cancer among younger men and the individualized use of PSA in an early detection setting.

Overall, while prostate cancer is a heritable disease at the individual level (Shevach and Cooney, 2025), current evidence suggests that disparities in mortality across Australasia are predominantly driven by differences in early detection practices, healthcare access, availability of advanced treatments, and disease management, underscoring the importance of targeted public health and clinical strategies aimed at improving awareness, early detection and treatment (James *et al*., 2024).

### Other major cancer sites

Stomach cancer, starting from extremely high rates, continues to markedly decline in Japan and the Republic of Korea, although predicted 2026 rates remain higher than those observed in Western countries. Across Eastern Asia, both *Helicobacter pylori* infection and gastric cancer burden have traditionally been substantially higher than in Western regions. Still, stomach cancer remains the third leading cause of cancer death in both sexes combined in Japan and the Republic of Korea. The population-based screening program in Japan, the implementation of organized gastric cancer screening in the Republic of Korea, and the declines in *H. pylori* prevalence across Asia have been associated with substantial reductions in gastric cancer mortality in recent decades (Nam, 2023; Sun *et al*., 2024; Cai *et al*., 2025). These programs have also improved early detection and survival, although mortality rates remain higher than those in Western populations (Kusano *et al*., 2026). Changes in diet including the reduction of salty and preserved foods (Morais *et al*., 2022), have also likely played a role. Hong Kong SAR, Israel, the Philippines, and Australia continue to exhibit substantially lower stomach cancer rates.

Lung cancer remains the leading cause of cancer death in most countries, with trends largely driven by historical smoking patterns (Berg *et al*., 2023; Collaborators, 2024). Female lung cancer mortality has surpassed that of breast cancer in Australia and Hong Kong SAR, mirroring patterns typically observed in Western countries and reflecting changes in smoking prevalence among subsequent generations of women in those countries. Female lung cancer rates in Hong Kong and Australia have started to decline over the last decades, reflecting reduced smoking prevalence and smoking cessation among females (AIHW,2025; Health Bureau. The Government of the Hong Kong Special Administrative Region of the People’s Republic of China, 2023). In other countries considered, female lung cancer mortality rates remain comparatively low (5–7/100 000) and show no consistent upward trends.

Colorectal cancer mortality was lower compared to Europe or North America. This can be attributed to historically lower exposure to Western lifestyle-related risk factors, including diets high in red and processed meat, obesity, and alcohol consumption. However, recent rates are comparable and, in most cases, lower than European or North American ones, reflecting upward trends in adoption of western diet and sedentary behaviour, alcohol consumption and obesity prevalence. These factors likely contributed to the reported rise in incidence, particularly among younger adults (Belay *et al.*, 2023; Global Obesity Observatory, 2025). Limited screening coverage in several Asian settings may also contribute to lower observed incidence but higher mortality-to-incidence ratios, reflecting later-stage diagnosis and treatment disparities (Fu *et al*., 2025).

Similarly to Europe, the USA, and most regions globally (Collaborators, 2019), mortality from pancreatic cancer is predicted to remain stable or increase in Japan, Hong Kong SAR and among women in Australia. This pattern contrasts with the more favourable trends observed for many other cancer sites and likely reflects the limited progress in early detection and treatment, together with the rising prevalence of obesity and metabolic disorders in these settings (He *et al.*, 2024).

Among women, breast cancer mortality in the Philippines is approximately double that observed in other Asian countries included in this study and represents the leading cause of predicted cancer deaths when both sexes are considered combined, making it the only country examined in which overall female cancer mortality rates are comparable to those of males. This cannot be totally attributable to westernization (Gibson *et al*., 2010) and reflects lower survival rates (Que *et al*., 2018; Yap *et al*., 2023). This pattern is further compounded by limited access to screening, up-to-date therapies, and optimal disease management (Ho *et al*., 2022; Jaya-Prakason *et al*., 2024). The rise in the Philippines until 2010 is likely, at least in part, due to improved diagnosis and certification of breast cancer and possibly reflects rise in incidence due to declining parity, later age at first birth, and increasing prevalence of obesity. Israel also showed higher mortality rates, consistent with its well documented higher prevalence of hereditary cancer-related mutations (Mahtani *et al*., 2023). No appreciable declines in breast rates were observed in the Republic of Korea, which however maintains the lowest breast cancer mortality.

Uterine cancer (including both endometrial and cervical cancers) shows unfavourable predicted trends in Hong Kong SAR, Japan, and Australia. These increases are largely driven by the rising burden of endometrial cancer, linked to the growing prevalence of obesity, metabolic comorbidities, population ageing, and declining parity in these countries (Morice *et al*., 2016; Yang *et al*., 2025). At the same time, improvements in cervical cancer control have been uneven: while Australia has long-standing screening programs and high HPV vaccination coverage (Australian Institute of Health and Welfare, 2023; Branda *et al*., 2024), Japan and Hong Kong SAR have low HPV vaccination uptake and variable screening participation (Hanley *et al*., 2015; Branda *et al*., 2024). As a result, combined uterine cancer mortality in these countries is projected to reach approximately 3.5–4.5 per 100 000 females. In contrast, although the Philippines shows more favourable predicted trends, Filipino women continue to exhibit the highest absolute mortality in the region (>7 per 100 000), reflecting persistent gaps in screening, HPV vaccination, and timely access to treatment (Ho *et al*., 2022; Branda *et al*., 2024).

Ovarian cancer mortality is projected to show less favourable trends in some Asian countries, including Hong Kong SAR, the Philippines, and Japan. Historically, the oral contraceptive use – the major protective factor for ovarian cancer (La Vecchia, 2017) – has been very low in Asia. In the Republic of Korea and Japan, the prevalence of oral contraceptives use among women aged 15–49 years was less than 4% in 2015 (Yoshida *et al*., 2016), likely contributing, at least in part, to the observed birth-cohort effects and the rising trends in ovarian cancer incidence (Wei *et al*., 2025). In addition, changes in reproductive patterns and a shift toward a westernized lifestyle and diet could also play a role (Chen *et al*., 2023; Bhattacharjee *et al*., 2024).

Bladder cancer mortality remains lower than in Europe and North America for both sexes (Filho *et al*., 2025), reflecting historically lower exposure to tobacco and occupational carcinogens, together with improvements in diagnosis and treatment, and potentially influenced by genetic differences between Australasian and European cohorts (Jubber *et al*., 2023). Tobacco alone, however, cannot explain the low rates reported in countries such as Hong Kong and Australia.

Mortality from leukaemias remains favourable across the region, with levels comparable to or lower than those observed in Europe and the USA (Ferlay *et al*., 2024). Australia and Japan, show the lowest rates, closely aligned with Western countries, consistent with the high availability of intensive chemotherapy, haematopoietic stem-cell transplantation, and targeted agents (Wu *et al*., 2025).

### Interpretation and implications

Caution is needed in the interpretation of cancer predictions. Our predictions assume that current trends in prevention, diagnosis, and treatment continue, and may not fully capture recent potential changes in healthcare policies, screening uptake, or emerging therapies. To limit random variability, we used data for 10 major cancer sites from relatively large populations with reliable death certification.

The persistent decline in age‑standardized mortality across most cancer sites reflects patterns of smoking cessation and other improvements in prevention, early detection, and treatment. However, the rising absolute number of deaths highlights the need for strengthened cancer control strategies, particularly in ageing populations. Prostate cancer exemplifies this challenge: although ASRs are declining in most countries, demographic ageing and rising incidence mean that its overall burden will remain substantial.

Cross‑country differences in prostate cancer mortality suggest that access to early diagnosis and modern treatments play a key role in shaping outcomes. Expanding equitable access to PSA‑based early detection strategies, improving diagnostic pathways, and ensuring availability of effective systemic therapies may contribute to further reductions, particularly in countries where mortality remains high.

In conclusion, despite persistent geographic variations, overall cancer mortality is projected to continue declining through 2026 in most Australasian countries. Prostate cancer shows favourable trends in several settings, but substantial disparities remain. Strengthening early detection and treatment pathways will be essential to sustain and accelerate progress in reducing prostate cancer mortality in the region.

## Acknowledgements

This work was supported by the AIRC Foundation for Cancer Research in Italy (Project no. 22987) and C.S. and C.L.V. were supported by EU funding within the Next Generation EU-MUR PNRR Extended Partnership initiative on Emerging Infectious Diseases (Project no. PE00000007, INF-ACT). The funding sources had no role in the design and conduct of the study; collection, management, analysis, and interpretation of the data; preparation, review, or approval of the manuscript; and the decision to submit the manuscript for publication. Additional support was provided by the Italian Ministry of Health Ricerca Corrente 5x1000 funds.

### Conflicts of interest

There are no conflicts of interest.
